# Vector analysis of corneal astigmatism in cataractous eyes based on IOLMaster 700 biometry

**DOI:** 10.1371/journal.pone.0300576

**Published:** 2024-04-19

**Authors:** Achim Langenbucher, Jascha Wendelstein, Alan Cayless, Peter Hoffmann, Nóra Szentmáry

**Affiliations:** 1 Department of Experimental Ophthalmology, Saarland University, Homburg/Saar, Germany; 2 Department of Ophthalmology, Johannes Kepler University Linz, Linz, Austria; 3 School of Physical Sciences, The Open University, Milton Keynes, United Kingdom; 4 Augen- und Laserklinik Castrop-Rauxel, Castrop-Rauxel, Germany; 5 Dr. Rolf M. Schwiete Center for Limbal Stem Cell and Aniridia Research, Saarland University, Homburg/Saar, Germany; 6 Department of Ophthalmology, Semmelweis-University, Budapest, Hungary; UNSW: University of New South Wales, AUSTRALIA

## Abstract

**Purpose:**

The purpose of this study was to investigate the effect of the corneal back surface by comparing the keratometric astigmatism (K, derived from the corneal front surface) of a modern optical biometer against astigmatism of Total Keratometry (TK, derived from both corneal surfaces) in a large population with cataractous eyes. The results were then used to define linear prediction models to map K to TK.

**Methods:**

From a large dataset containing bilateral biometric measurements (IOLMaster 700) in 9736 patients prior to cataract surgery, the total corneal astigmatism was decomposed into vectors for K, corneal back surface (BS), and TK. A multivariate prediction model (MV), simplified model with separation of vector components (SM) and a constant model (CM) were defined to map K to TK vector components.

**Results:**

The K centroid (X/Y) showed some astigmatism with-the-rule (0.1981/-0.0211 dioptre (dpt)) whereas the TK centroid was located around zero (-0.0071/-0.0381 dpt against-the-rule) and the BS centroid showed systematic astigmatism against-the-rule (-0.2367/-0.0145 dpt). The respective TK–K centroid was located at -0.2052/-0.0302 dpt. The MV model showed the same performance (i.e. mean absolute residuum) as the SM did (0.1098 and 0.1099 dpt respectively) while the CM performed only slightly worse (0.1121 dpt mean absolute residuum).

**Conclusion:**

In cases where tomographic data are unavailable statistical models could be used to consider the overall contribution of the back surface to the total corneal astigmatism. Since the performance of the CM is sufficiently close to that of MV and SM we recommend using the CM which can be directly considered e.g. as surgically induced astigmatism.

## Background

In addition to ocular distances such as axial length (AL), anterior chamber depth (ACD, measured from corneal epithelium to the front vertex of the crystalline lens), or central thickness of the cornea (CCT) or crystalline lens (LT), the corneal power is known to have the largest impact on the prediction of the power of the intraocular lens implant (IOL) [1]. In general, corneal power cannot be measured *in situ*, but modern corneal or anterior segment tomographers based on Scheimpflug slit projection techniques or optical coherence tomography (OCT) enable clinicians to derive the curvatures of the corneal front and back surfaces and the corneal thickness profile with high precision [1]. In contrast to classical manual or automated keratometers which are restricted to deriving the corneal radius of curvature at a number of conjugate points in the mid periphery, Placido topographers can measure the curvature profile of the entire corneal front surface with a high precision [1–3]. However, both technologies require a sufficient specular reflex from the pre-corneal tearfilm, making measurements unreliable or impossible in situations with tearfilm pathologies [4]. In contrast, Scheimpflug or OCT tomographers analyse the diffuse volume scattering of the cornea making these devices mostly independent from the tearfilm status [1].

Toric IOLs have gained in popularity over the last 2 decades, mostly because of premium lenses designed to recover some depth of field in pseuophakic patients appearing on the market. For those concepts of depth of field lenses such as monofocal plus, enhanced depth of focus (EDOF) or bifocal /multifocal lenses, a correction of the residual corneal astigmatism is mandatory, and indications for toric IOLs currently start at a corneal astigmatism as low as 0.75 dioptre (dpt). Today, more or less all IOL manufacturers provide their lens models with and without correction of corneal astigmatism, starting with a toric correction of 1 dpt.

Since Javal it is well known that keratometers typically do not provide a complete measure of corneal astigmatism [5]. Specifically, the corneal back surface adds some amount of against-the-rule astigmatism which is not reflected in the measurement of the corneal front surface. In with-the-rule astigmatism of the corneal front surface, the total corneal astigmatism is typically lower than the measured keratometric astigmatism, whereas in against-the-rule astigmatism of the corneal front surface the total corneal astigmatism is typically larger than the keratometric astigmatism [5]. With oblique axes, the amount and direction of total corneal astigmatism might differ from the keratometric astigmatism [6].

In the literature we find different philosophies on how to derive corneal astigmatism for calculation of toric IOLs: some clinicians prefer to measure keratometry and consider the contribution of the corneal back surface to the total corneal astigmatism using a statistical prediction model [6], whereas others prefer a direct measurement of the corneal curvature in both surfaces and a calculation of the total corneal astigmatism using crossed cylinders [2, 3]. However, a decomposition of astigmatism into vector components is appropriate in all cases [6]. In this context, corneal power as clinically described in terms of sphere, cylinder and axis has to be transformed in 3-dimensional vector components with equivalent power’ and the astigmatic projections to the horizontal and vertical (0° and 90°) and the oblique (45° and 135°) meridians [7].

In addition to the corneal front and back surface measurements, most modern corneal or anterior segment tomographers provide a composite value which describes the refractive power of the entire cornea considering the corneal front and back surfaces [8]. These composite values are recommended by some researchers for use in toric lens power calculation instead of a direct use of keratometric readings considering the corneal front surface only with or without a statistical prediction model [9].

The goals of the present study are to analyse keratometric, corneal back surface, and total corneal astigmatism vectors based on a large dataset of bilateral measurements made with a modern optical biometer prior to cataract surgery, and to derive simple prediction models for the contribution of the corneal back surface astigmatism to the total corneal astigmatism by comparing the keratometric and total corneal astigmatism.

## Methods

### Dataset for our analysis

We used a dataset containing a total of 19,472 biometrical measurements (bilateral measurements of 9,736 patients made on the same examination day) using the IOLMaster 700 (Carl-Zeiss-Meditec, Jena, Germany) from one clinical centre (Augenklinik Castrop, Castrop-Rauxel, Germany) for this retrospective study. All measurements were performed in cataract patients, excluding pseudophakic eyes. Duplicate measurements of eyes, eyes in pharmacologically stimulated mydriasis (pupil width more than 5.2 mm), and incomplete records in the dataset had already been discarded at the source. Measurements made after refractive surgery were also excluded from the dataset at source, as were those indicated as having ectatic corneal diseases (keratoconus, keratoglobus, pellucid marginal degeneration) or other corneal pathologies. The data were transferred to a.csv data table using the data export module of the IOLMaster 700 software. Data tables were reduced to the relevant parameters required for our data analysis, consisting of: laterality (left or right eye), patient’s age at the time of examination, keratometry (K) (curvature of the corneal front surface in the flat (FR1) and the steep (FR2) meridian both in mm together with the axis of the flat meridian (FA1)), corneal back surface measurement (BS)(curvature of the corneal back surface in the flat (BR1) and the steep (BR2) meridian both in mm together with the axis of the flat meridian (BA1)), Total Keratometry (TK)(curvature in the flat (TKR1) and the steep (TKR2) meridian both in mm together with the axis of the flat meridian (TKA1)), axial length of the eye (AL in mm), central corneal thickness (CCT in mm), anterior chamber depth (ACD) measured from the corneal front apex to the crystalline lens front apex in mm, and the central thickness of the crystalline lens (LT in mm). The data were transferred to Matlab (Matlab version 2019b, MathWorks, Natick, USA) for further processing.

All procedures performed in this study were in accordance with the ethical standards of the Ärztekammer des Saarlandes and with the 1964 Helsinki declaration and its later amendments or comparable ethical standards. The local ethics committee (IRB) has provided a waiver for this study (Ärztekammer des Saarlandes, 157/21), as all data processed in this study were already anonymized at the source before being transferred to us (November 03, 2022) for processing. This precludes any back-tracing of the identity, and therefore informed consent of the patients was not necessary.

### Preprocessing of the data

Custom software for data processing and analysis was written in Matlab. From the curvature and axis data of K, BS and TK (K: (FR1, FR2, FA1; BS: BR1, BR2, BA1; TK: TKR1, TKR2, TKA1)) we derived the respective power vector components as: (K: FSEQ = 0.5 (332/FR1+332/FR2), FC0 = (332/FR2-332/FR1) cos(2·FA1), FC45 = (332/FR2-332/FR1) sin(2·FA1); BS: BSEQ = -0.5 (40/BR1+40/BR2), BC0 = (40/BR2-40/BR1) cos(2·BA1), BC45 = (40/BR1-40/BR2) sin(2·BA1); TK: TKSEQ = 0.5 (332/TKR1+332/TKR2), TKC0 = (332/TKR2-332/TKR1) cos(2·TKA1), TKC45 = (332/TKR2-332/TKR1) sin(2·TKA1)) based on a keratometer index n_K_ = 1.332 and refractive indices for cornea n_C_ = 1.376 and aqueous humour n_A_ = 1.336 derived from the Liou-Brennan schematic model eye [10]. Since the calculation strategy for Total Keratometry in the IOLMaster 700 software is undisclosed, we calculated the power vector components for the corneal front vertex power (FV)(equivalent power FVSEQ and projections of the astigmatism to the 0/90° FVC0 and 45°/135° meridian FVC45) as a representation of total corneal power at the corneal front vertex plane using a calculation scheme for toric vergences as described in previous papers [7, 8].

The dataset was split into left (OS) and right (OD) eyes. To account for the mirror symmetry between left and right eyes, the Y components of the power vectors (FC45, BC45, TKC45, and FVC45 components) for K, BS, TK and FV were reversed in sign for left eyes (OS) to consider all eyes in the study as right eyes (OD) [11, 12]. The astigmatism vector components were studied both for the OS and OD data separately and for the pooled data.

### Statistics and linear prediction model for ocular magnification

The biometric data (AL, CCT, ACD and LT) and the power vector components of K, BS, TK and FV are shown descriptively in terms of mean (MEAN), standard deviation (SD), median (MEDIAN) as well as the lower and upper boundaries of the 95% confidence interval (2.5% and 97.5% quantile) for the entire dataset and separately for OS and OD. Data for K, BS, TK and FV are displayed using double angle plots where the projections of the astigmatic components to the 0°/90° meridian and the 45°/135° meridian are plotted on the X and Y axes [5, 6]. The astigmatic power vector components were tested for bivariate normality using the Henze-Zirkler Test for multivariate paired samples [13–15], and differences between astigmatic power vector components of TK and FV were analysed using the Hotelling’s T^2^ Test [16] (where bivariate data are normally distributed) or the Sign Rank Test for multivariate paired samples [17, 18] (where bivariate data are non-normally distributed).

As a simplification, assuming bivariate normal distributions for the astigmatic power vector components, 95% error ellipses were calculated based on the covariance matrix and the ellipse area and the centroid were derived [7]. In addition, the 95% confidence region was calculated without assumptions of parametric distributions by iteratively eroding the data clouds according to their convex hull envelope (iterative convex hull stripping) [19, 20], and the area of the 95% confidence region and the spatial median (medoid) were derived [20–22].

For generating a prediction model to map the astigmatism vector components K to all eyes were considered as right eyes as outlined before [7, 11]. Various models (multivariate (bivariate) linear regression using the ECM algorithm [23], univariate linear regression with a separation of the 2 vector components, and constant model) were defined and analysed in their performance [24].

## Results

For our analysis 9736 right and 9736 left eyes from 9736 patients (N = 5,492 female and 4,243 male patients) were enrolled in our study. The mean age of the study population was 69.26±15.39 years (median 72.58 years, 95% confidence interval from 44.20 to 87.61 years). The mean AL was 23.67±1.40 mm (median 23.49 mm, 95% confidence interval from 21.52 to 27.06 mm), mean CCT was 552±36.8 μm (median 551 μm, 95% confidence interval from 482 to 626 μm), mean ACD was 3.13±0.42 mm (median 3.13 mm, 95% confidence interval from 2.33 to 3.97 mm), and mean LT was 4.61±0.49 mm (median 4.64 mm, 95% confidence interval from 3.42 to 5.47 mm). The descriptive data for the power vectors for K, BS, TK and FV for the entire dataset and for the left and right eyes are listed in **Table 1**.

**Table 1:** Explorative data of power vectors derived from the IOLMaster 700 for the entire population and for left and right eyes. For each parameter (..)SEQ refers to the equivalent power, and (..)C0 and (..)C45 to the projections of the astigmatism to the 0°/90° and 45°/135° meridins respectively. The table shows the arithmetic mean (Mean), standard deviation (SD), median (Median) and the lower and upper boundaries of the 95% confidence interval (2.5% and 97.5% quantiles).

Power vector, data in dpt		Keratometry K			Back Surface measurement BS			Total Keratometry TK			Front Vertex power FV		
		FSEQ	FC0	FC45	BSEQ	BC0	BC45	TKSEQ	TKC0	TKC45	FVSEQ	FVC0	FVC45
Entire dataset, N = 19,472	Mean	43.1085	0.1981	-0.0211	-6.8327	-0.2367	-0.0145	43.1706	-0.0071	-0.0381	43.1801	-0.0032	-0.0379
	SD	1.5123	0.9644	0.5743	0.2387	0.1509	0.1070	1.5170	0.9948	0.5907	1.5187	0.9962	0.5915
	Median	43.0822	0.1541	-0.0147	-8275	-0.2315	-0.0133	43.1375	-0.0348	-0.0326	43.1471	-0.0426	-0.0498
	2.5% quantile	40.2099	-1.6139	-1.1182	-6.3047	-0.5555	-0.2238	40.2558	—1.8765	-1.1632	40.2628	-1.8755	-1.1641
	97.5% quantile	46.1014	2.3227	1.0000	-5.3809	0.4444	0.1910	46.1706	2.1624	1.1208	46.1796	2.1742	1.1249
OS, N = 9,736	Mean	43.1309	0.2080	0.0063	-6.8393	-0.2426	0.0126	43.1896	-0.0016	0.0196	43.1992	0.0024	0.0193
	SD	1.5169	0.9686	0.5826	0.2394	0.1521	0.1032	1.5212	0.9984	0.5965	1.5228	0.9998	0.5974
	Median	43.1014	0.1609	0.0061	-6.8344	-0.2357	0.0087	43.1520	-0.0309	0.0180	43.1609	-0.0402	0.0270
	2.5% quantile	40.2403	-1.5922	-1.1564	-6.3087	-0.5689	-0.1791	40.2861	-1.8719	-1.1546	40.2879	-1.8703	-1.1569
	97.5% quantile	46.1280	2.3697	1.0929	-4.3921	0.0420	0.2132	46.2015	2.1994	1.1431	46.2179	2.2056	1.1446
OD, N = 9.736	Mean	43.0862	0.1882	-0.0485	-6.8262	-0.2308	-0.0416	43.1516	-0.0127	-0.0957	43,1609	-0.0088	-0.0952
	SD	1.5075	0.9602	0.5647	0.2377	0.1495	0.1039	1.5127	0.9912	0.5791	1.5144	0.9925	0.5799
	Median	43.0630	0.1460	-0.0554	-5.8205	0.2275	-0.0383	43.1215	-0.0382	-0.1029	43.1302	-0.0459	-0.1031
	2.5% quantile	40.1966	-1.6316	-1.0968	-6.2969	-0.5408	-0.2508	40.2381	-1.8813	-1.1698	40.2428	-1.8824	-1.1690
	97.5% quantile	46.0627	2.2758	1.1047	-5.3721	0.0461	0.1516	46.1275	2.1315	1.0916	46.1476	2.1396	1.0939

**Fig 1:**
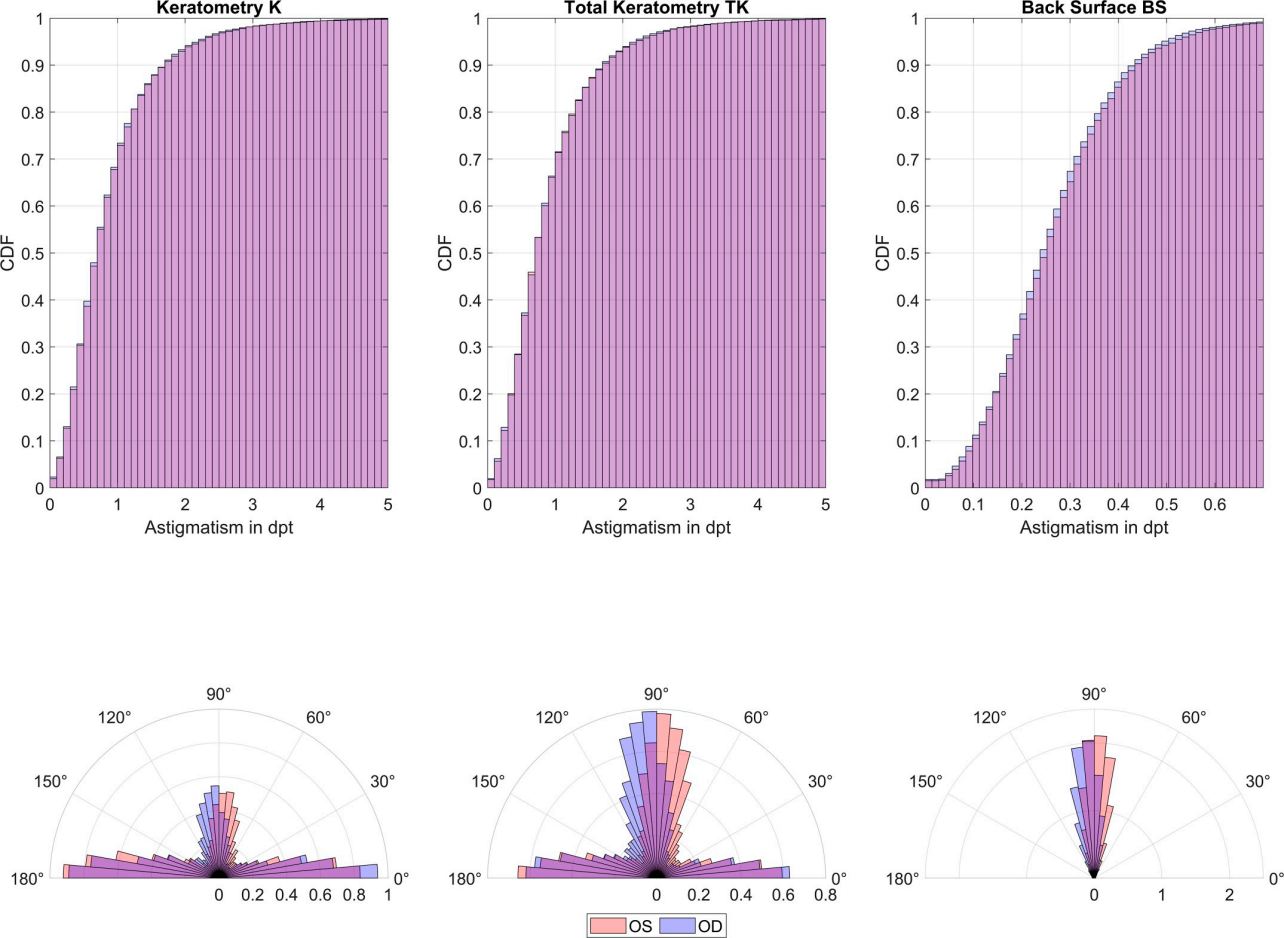
Upper row: cumulative density plots (CDF) of the absolute value of astigmatism for Keratometry K (left graph), Total Keratometry TK (middle graph), and Back Surface BS (right graph) for left eyes (OS, red) and right eyes (OD, blue). Lower row: polar histogram showing the axis distributions of the flat axis for K, TK, and BS for OS and OD. Please note that for the polar histograms only astigmatism values with a radius difference between flat and steep meridian of ≥0.1 mm were considered.

The upper row of **Fig 1** displays the cumulative density plots (CDF) of the absolute value astigmatism for K (left graph), TK (middle graph), and BS (right graph) for left eyes (OS, red) and right eyes (OD, blue). In the lower row the respective polar histograms are provided. These histograms show the distribution of the flat K, TK, and BS axes for left and right eyes for all cases with a difference of at least 0.1 mm between the radii in the flat and steep meridians.

**Fig 2:**
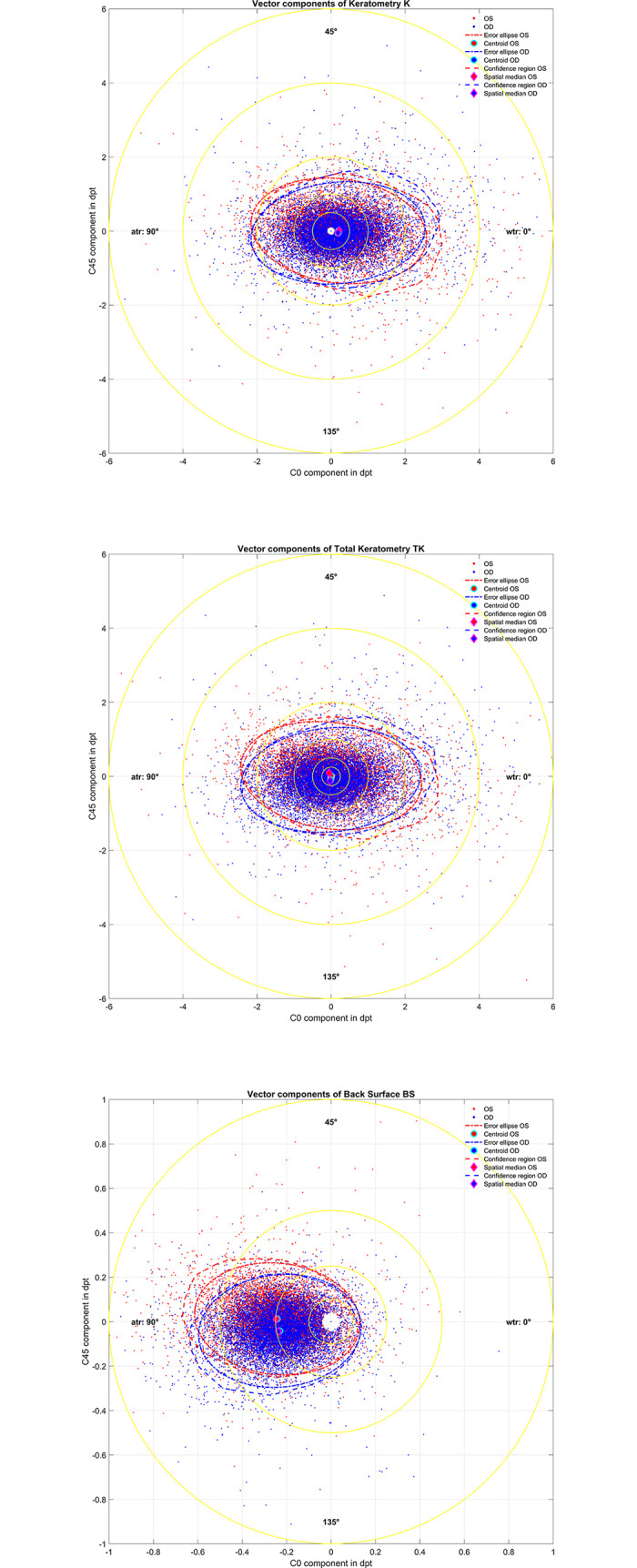
Double angle plots for keratometry (K, upper graph), Total Keratometry (TK, middle graph), and Back Surface (BS, lower graph) astigmatism for left eyes (red dots) and right eyes (blue dots). 95% error ellipses (red and blue dash-dotted lines) derived from the covariance matrix are displayed together with the centroids (red and blue filled circle), and the 95% confidence regions (red and blue dashed lines) derived from an iterative convex hull stripping procedure are shown together with the spatial medians (red and blue filled rhombi).

**Fig 2** shows the double angle plots for keratometry (K, upper graph), Total Keratometry (TK, middle graph), and Back Surface (BS, lower graph) astigmatism for left and right eyes together with the respective 95% error ellipses derived from the covariance matrix and centroids, and the 95% confidence regions derived with an iterative convex hull stripping procedure and the spatial median of the bivariate astigmatic power vectors. The respective location of the centroid and the area of the 95% error ellipse and the location of the spatial median and the 95% confidence region are listed in **Table 2**.

**Table 2:** Descriptive data of the centroid location and the area of the 95% error ellipse derived from the covariance matrix together with the spatial median location and the area of the 95% confidence region derived from iterative convex hull stripping. Data are shown for Keratometry K, Total Keratometry TK, Back Surface measurement BS, and for the differences TK–K and Front Vertex power FV-K for left eyes (OS) and right eyes (OD). The last 2 rows of the table show the respective data for the differences TK–K and FV–K for all eyes. Here the C45 vector components of left eyes were reversed in sign considering symmetry conditions.

N = 19,472 eyes 9,735 OS 9,736 OD	Eye	Centroid X in dpt	Centroid Y in dpt	Area of 95% error ellipse in dpt^2^	Spatial median X in dpt	Spatial median Y in dpt	Area of 95% confidence region in dpt^2^
Keratometry K	OS	0.2080	0.0063	10.5173	0.2149	0.0123	12.3980
	OD	0.1882	-0.0485	10.1452	0.1853	-0.0423	12.1845
Total Keratometry TK	OS	-0.0016	0.0196	11.0825	-0.0546	0.0959	13.1409
	OD	-0.0127	-0.0957	10.7293	-0.0286	-0.1229	12.6228
Back Surface BS	OS	-0.2426	0.0126	0.2842	-0.2475	0.0116	0.3235
	OD	-0.2308	-0.0416	0.2918	-0.2364	-0.0440	0.3195
TK–K	OS	-0.2096	0.0132	0.1458	-0.2092	0.0113	0.1453
	OD	-0.2008	-0.0472	0.1514	-0.2010	0.0471	0.1475
FV–K	OS	-0.2056	0.0130	0.1406	-0.2039	0.0126	0.1409
	OD	-0.1996	-0.0467	0.1459	-0.1963	-0.0468	0.1444
TK–K OS and OD		-0.2052	-0.0302	0.1527	-0.2065	-0.0295	0.1531
FV–K OS and OD		-0.2013	-0.0299	0.1474	-0.2024	-0.0296	0.1461

**Fig 3:**
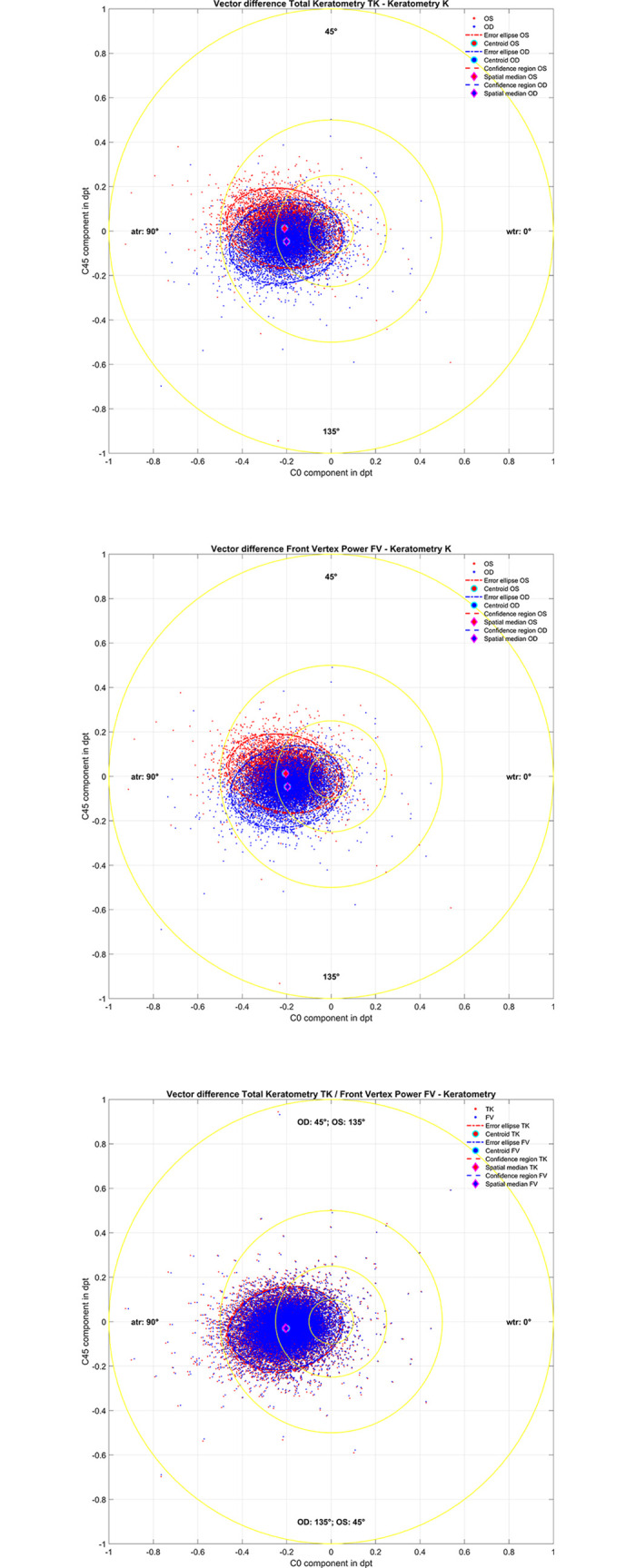
Double angle plots for the difference Total Keratometry TK–Keratometry K for left and right eyes (upper graph), Front Vertex power FV–Keratometry K for left and right eyes (middle graph), and the differences Total Keratometry TK–Keratometry K and Front Vertex power FV–Keratometry K for all eyes after considering the symmetry of left and right eyes (lower graph). In the upper and middle graph the left and right eye data are shown in red and blue, and in the lower graph the differences TK–K and FV–K are displayed in red and blue. 95% error ellipses (dash-dotted lines) derived from the covariance matrix are displayed together with the centroids (filled circles), and the 95% confidence regions (dashed lines) derived from an iterative convex hull stripping procedure are shown together with the spatial medians (filled rhombi). Please note that in the lower graph the axis for right eyes turns counterclockwise and the axis for left eyes turns clockwise.

In **Fig 3** the double angle plots for the difference TK–K (upper graph) and FV–K (middle graph) are displayed for left and right eyes. In the lower graph the differences TK–K and FV–K are shown for all eyes after considering the symmetry of left and right eyes. The 95% error ellipses in the graphs (dash-dotted lines) as derived from the covariance matrix are displayed together with the centroids (filled circles), and the 95% confidence regions (dashed lines) derived from an iterative convex hull stripping procedure. The spatial medians (filled rhombi) are also shown. In the lower graph the axis for right eyes turns counterclockwise and the axis for left eyes turns clockwise. The respective locations of the centroid and the area of the 95% error ellipse and the locations of the spatial median and the 95% confidence region are listed in **Table 2**.

The astigmatic difference data TK–K and FV-K do not follow a bivariate normal distribution for OS, OD, and for both eyes (taking into account the reflection of OS values to match OD)(Henze-Zirkler, p<0.001). The Sign Rank Test for multivariate paired samples showed a highly significant difference between bivariate K and TK and between K and FV in both subsets (OS and OD), and in the combined dataset for both eyes (again taking account of the reflection of the OS data) (P < 0.001). The difference data of OD and OS of TK–K and FV–K also did not follow a bivariate normal distribution (Henze-Zirkler, p<0.01). The Sign Rank Test for multivariate paired samples showed no significant difference between the (reflected) OS and OD subsets and also no significant difference for either TK–K or FV—K (P = 0.0625).

The multivariate linear model which maps Keratometry K to Total Keratometry TK for all eyes (considering OS eyes as OD) reads:

$${\left[\begin{array}{c} TKC0\\ TKC45 \end{array}\right]}_{\mathrm{predicted}}=\left[\begin{array}{cc} 1.0257 & 0.0038\\ 0.0052 & 1.0164 \end{array}\right]∙\left[\begin{array}{c} FC0\\ FC45 \end{array}\right]+\left[\begin{array}{c} -0.2102\\ -0.0308 \end{array}\right]$$

with a log likelihood objective function at the last iteration of logL = 3.921·10^4^ and a mean residuum error of 0.1098 dpt. A simplified version of this regression without interaction of the 2 astigmatic vector components reads:

$${\left[\begin{array}{c} TKC0\\ TKC45 \end{array}\right]}_{\mathrm{predicted}}=\left[\begin{array}{c} 1.0260\\ 1.0174 \end{array}\right]∙\left[\begin{array}{c} FC0\\ FC45 \end{array}\right]+\left[\begin{array}{c} -0.2104\\ -0.0297 \end{array}\right]$$

with a mean residuum of 0.1099 dpt. The constant model describing the difference with an intercept vector reads:

$${\left[\begin{array}{c} TKC0\\ TKC45 \end{array}\right]}_{\mathrm{predicted}}=\left[\begin{array}{c} FC0\\ FC45 \end{array}\right]+\left[\begin{array}{c} -0.2052\\ -0.0302 \end{array}\right]$$

with a mean residuum error of 0.1121 dpt.

The multivariate linear model which maps Keratometry K to Front Vertex power FV for all eyes (considering OS eyes as OD) reads:

$${\left[\begin{array}{c} FVC0\\ FVC45 \end{array}\right]}_{\mathrm{predicted}}=\left[\begin{array}{cc} 1.0273 & 0.0038\\ 0.0051 & 1.0182 \end{array}\right]∙\left[\begin{array}{c} FC0\\ FC45 \end{array}\right]+\left[\begin{array}{c} -0.2066\\ -0.0304 \end{array}\right]$$

with a log likelihood objective function at the last iteration of logL = 4.005·10^4^ and a mean residuum error of 0.1075 dpt. A simplified version of this regression without interaction of the 2 astigmatic vector components reads:

$${\left[\begin{array}{c} FVC0\\ FVC45 \end{array}\right]}_{\mathrm{predicted}}=\left[\begin{array}{c} 1.0276\\ 1.0193 \end{array}\right]∙\left[\begin{array}{c} FC0\\ FC45 \end{array}\right]+\left[\begin{array}{c} -0.2067\\ -0.0293 \end{array}\right]$$

with a mean residuum error of 0.1076 dpt. The constant model describing the difference with an intercept vector reads:

$${\left[\begin{array}{c} FVC0\\ FVC45 \end{array}\right]}_{\mathrm{predicted}}=\left[\begin{array}{c} FC0\\ FC45 \end{array}\right]+\left[\begin{array}{c} -0.2013\\ -0.0299 \end{array}\right]$$

with a mean residuum error of 0.1101 dpt.

## Discussion

In this study, three linear prediction models for mapping keratometric astigmatism K to total keratometry TK were developed, based on a dataset of IOLMaster 700 measurements. For comparison K was also mapped to a front vertex power FV calculated from measures of both corneal surfaces.

The main findings were that FV did not show any clinically relevant differences from TK, and that all three models showed similar performances. The performances of the univariate and multivariate models were almost the same, with the constant model showing only a very slightly reduced performance, sufficient to be used in clinical routine where tomographic measurements of total corneal astigmatism are unavailable.

The increasing popularity of toric intraocular lenses in cataract surgery highlights the need for accurate measurements of corneal astigmatism. In most cases the power of toric lenses is calculated based on the measurement of corneal front surface curvature from a keratometer integrated in the optical biometer, even though some modern Scheimpflug or OCT based biometers provide both corneal front and back surface curvature data [7]. As a rule of thumb that in with-the-rule corneal astigmatism toric lenses calculated with keratometric readings tend to overcorrect corneal astigmatism and in against-the-rule astigmatism they tend to undercorrect corneal astigmatism [5]. Several statistical correction schemes have been developed in an attempt to correct the centroid error between postoperative refractive cylinder considered at the corneal plane and keratometric astigmatism [9] or between Total Keratometry astigmatism and keratometric astigmatism. However, the measures of corneal back surface curvature in particular may vary a lot between tomographers since the raw data generally have to be corrected using inverse raytracing through the corneal front surface [8].

Using anterior segment tomographers or last generation biometers the geometry of both corneal surfaces can be derived with a good precision [1, 2], but most of the intraocular lens power formulae are unable to deal with a thick lens model of the cornea with two surfaces and crossed cylinders or with total corneal power data composed from both surfaces [9]. Therefore statistical corrections for the contribution of corneal back surface astigmatism, which is not reflected by standard keratometry, are of high relevance in clinical practice even taking into account that there is a large individual scatter of corneal back surface curvature and astigmatism which cannot be addressed with such correction models [7, 9].

The purpose of this paper was to investigate the measured corneal front surface curvature, back surface curvature, and TK from a modern optical biometer in a large dataset with cataractous eyes where measurements of both eyes were available. Since the calculation strategy of TK measures from corneal front and back surface curvature is not disclosed, we derived the corneal FV power as a measure for a thin lens equivalent [8] describing the refraction of the cornea with respect to the corneal front vertex plane. This means that the cornea as meniscus lens could be replaced by FV located in a plane which is commonly used as the origin for all biometric distances [8]. From the clinical data we derived a linear prediction model which maps the corneal front surface measurement in terms of K to TK and also to FV.

Our results indicate that the overall mean C0 component of keratometric astigmatism ranges around 0.20 dpt (95% confidence interval: -1.6 to 2.3 dpt) whereas the C45 component ranges around -0.02 dpt (95% confidence interval: -1.1 to 1.0 dpt) with a slight trend to positive values for OD and negative values for OS. The overall mean C0 component of corneal back surface astigmatism ranges around -0.24 dpt (95% confidence interval: -0.6 to 0.4 dpt) whereas the C45 component again ranges around -0.01 dpt (95% confidence interval: -0.2 to 0.2 dpt) with a slight trend to negative values for OD and positive values for OS. The Total Keratometry value provided by the IOLMaster 700 represents the cornea using a thin lens model and shows overall mean values for the C0 / C45 component of around 0.00 dpt (95% confidence interval: -1.9 to 2.2 dpt) / -0.03 (95% confidence interval: -1.2 to 1.1 dpt). These values are very similar to the Front Vertex power values derived in this study to represent the thin lens model replacement at the corneal front vertex plane (C0 / C45 component of around 0.00 dpt (95% confidence interval: -1.9 to 2.2 dpt) / -0.04 (95% confidence interval: -1.2 to 1.1 dpt)).

The polar angle histograms in **Fig 1** present the probability density function of astigmatism axes (axis of the flat meridian) for K, TK and BS. The left plot (K) shows a much higher prevalence of keratometric astigmatism with-the-rule than against-the-rule, and it can also be seen that oblique axes are quite rare in our dataset. On the other hand, the plot for Total Keratometry TK shows roughly equal prevalences of astigmatic axes with-the-rule and against-the-rule, while oblique astigmatism axes again are quite rare. However, the graphs in the lower row show that there is some symmetry between left and right eyes for K, TK, and BS where right / left eyes with against-the-rule astigmatism tend to have their flat meridian at angles slightly lower / higher than 90° [5, 12]. This difference between K and TK astigmatism results mostly from corneal back surface astigmatism which are against-the-rule for nearly all eyes. The symmetry between left and right eyes is again obvious where right / left eyes tend to have their flat meridian at angles slightly lower / higher than 90° respectively.

The double angle plots in **Fig 2** indicate a large scatter of corneal astigmatism for K, TK, and BS in both the C0 and C45 components. For very small values of corneal astigmatism the the IOLMaster 700 sets the values systematically to zero (white spot in all 3 graphs in the center). The centroids describing the bivariate mean of the scatter coincide closely for both OS and OD as do the spatial median values describing the center of mass with respect to the Euclidian distances. On the other hand, the 95% error ellipses extracted from the covariance matrix (parametric setup) and the 95% confidence regions derived by an iterative convex hull stripping [19] (nonparametric setup) show some systematic differences. These differences result mostly from the non-normality of the bivariate astigmatism components. For K and TK the distribution seems to be somewhat tailed towards positive C0 values again with symmetry of left / right eyes in the C45 component, whereas for BS the distribution seems to be somewhat tailed towards negative C0 values. This finding suggests that in future it might be beneficial to analyse the bivariate distribution of the astigmatism vector to assess whether the error ellipses together with the centroid or any type of error region (e.g. derived from an iterative convex hull stripping [19]) together with the spatial median should be displayed.

In the double angle plots (**Fig 3**) the difference TK—K (upper graph) and the difference FV—K (middle graph) are shown for the left and the right eyes. Here the 95% error ellipses show a much better coincidence with the 95% error regions and the centroids fully match the spatial medians, indicating that the bivariate normal distributions are more symmetrical (even if they prove to be non-normal). For TK–K and FV–K there is some symmetry between left eyes and right eyes in the vertical direction (with respect to C0 = 0) which rectifies a common prediction model for both eyes for a mapping of K to TK or to FV. The lower graph in **Fig 3** displays the differences TK–K and FV–K for all eyes after considering all eyes as right eyes. The error ellipses and error regions for TK–K and FV–K show a very good coincidence as do the centroids and medoids. For OD / OS the flat axes of TK–K and FV–K are slanted slightly to values larger / smaller than 90° respectively.

In summary, C45 vector components reversed in sign to account for the intrinsic mirror symmetry between left and right eyes, a good correspondence was found between OD and OS distributions, indicating bilateral symmetry of these corneal parameters in most individuals. Based on this symmetry, we developed and tested 3 versions of linear prediction models to map K to TK and K to FV: first, a multivariate model based on the ECM algorithm [23, 24] in which both vector components C0 and C45 of K are considered as predictors for both components C0 and C45 of TK or FV. Second, simplified version or linear prediction model we assumed that C0 of K only affects C0 of TK or FV and C45 of K only affects C45 of TK or FV. This model follows the idea of Abulafia et al. who developed a linear prediction model derived from postoperative refraction and preoperative keratometry separately for both vector components C0 and C45 [9]. Third, we simplified our prediction model to a constant model in which the mapping of K to TK or FV is described by an intercept. The constant model might provide a simple but clinically powerful correction for K values if no software implementation of the multivariate regression is available. The multivariate model and simplified model show identical performance in terms of the mean Euclidian norm of the residuum (0.1098 vs. 0.1099 dpt and 0.1075 vs. 0.1076 dpt for mapping K to TK and to FV), and the constant model performs only marginally worse (0.1121 and 0.1101 dpt). To map K to TK or FV an astigmatism of 0.2013 or 0.2052 dpt against-the-rule has to be added, and the very small C45 intercept (-0.302 or -0.0299) can be ignored for clinical purposes. If the calculation scheme for toric IOLs allows such a statistical correction for the corneal back surface astigmatism, the necessary values could be entered directly in the software. Alternatively this could be considered as ‘surgically induced astigmatism SIA’. If the multivariate or the simplified linear regression is used in clinical practice we have to note that for left eyes both off-diagonal elements in the matrix (multivariate model) and the second element in the intercept vector (multivariate and simplified linear model) must be reversed in sign.

This study has some limitations: firstly, the data are derived from the IOLMaster 700 biometer and cannot be directly generalised to other biometers. Since the IOLMaster is available with or without the TK function such a statistical correction for the corneal back surface astigmatism might be relevant for clinicians who do not have access to the TK measurement. Secondly, we restricted the analysis to the effect of corneal back surface astigmatism in our correction models. Additional potential effects such as IOL displacement or tilt which could also affect the postoperative refractive cylinder were not considered as part of this study. Finally, **Fig 3** shows a large individual scatter in TK–K and FV–K which makes prediction of the total corneal astigmatism difficult with any model.

In **conclusion**, all three of the prediction models considered in this study (univariate, multivariate and constant) showed similar levels of performance in terms of mean absolute residuum. While the constant model was slightly behind the other two, its performance was still sufficient for clinical use where total corneal astigmatism data are not available.

Since the constant model (0.2 dpt against-the-rule) is simpler to implement in the clinical setting as compared to the more complex bivariate and univariate models, we recommend the constant model for general clinical use.

## Abbreviations

(.)C0: Vector component Vector component of astigmatism, projection to the 0° / 90° meridian
(.)C45: Vector component Vector component of astigmatism, projection to the 45° / 135° meridian
(.)SEQ: Spherical equivalent Average power of a refractive surface
[.]_predicted_: [.] output predicted by a linear model
ACD: Anterior chamber depth Distance from corneal front vertex to lens front vertex as measured by biometry
AL: Axial length Axial distance of the eye from corneal front vertex to the retina as measured by biometry
B(.): BR1/BR2, BA1/BA2 Radius of curvature of the corneal back surface in the flat / steep meridian in mm, orientation of the flat / steep meridian in
BS: Back surface power Refractive power of the corneal back surface
CCT: Central corneal thickness Central thickness of the cornea from epithelium to endothelium as measured by biometry
CM: Constant model Prediction model which considers a constant offset for each target parameter
dpt: dioptre Refractive power in 1/m
ECM: Algorithm Expectation–maximization(EM) algorithm is an: iterative method: to find: maximum likelihood: or: maximum a posteriori: estimates of: parameters: in: statistical models
F(.): FR1/FR2, FA1/FA2 Radius of curvature of the corneal front surface in the flat / steep meridian in mm, orientation of the flat / steep meridian in
FV: Front Vertex Power Refractive power of a thin replacement lens for the cornea positioned at the corneal front vertex plane
K: Keratometry Measurement of the corneal front surface curvature
logL: Objective function Log likelihood objective function at the last iteration, used as performance metric for the ECM algorithm
LT: Lens thickness Central thickness of the lens from front to back vertex as measured by biometry
MV: Multivariate model Prediction model which considers >1 predictors together with a constant offset for each target parameter
OD: Right eye
OS: Left eye
SM: Simplified model Univariate prediction model which considers a single predictor together with a constant offset for each target parameter
T(.): TKR1/TKR2, TKA1/TKA2 Radius of curvature of TK in the flat / steep meridian in mm, orientation of the flat / steep meridian in
TK: Total Keratometry Composite value provided by the IOLMaster 700 to describe the total corneal power
